# Genome-wide DNA methylation profiling with MeDIP-seq using archived dried blood spots

**DOI:** 10.1186/s13148-016-0242-1

**Published:** 2016-07-26

**Authors:** Nicklas H. Staunstrup, Anna Starnawska, Mette Nyegaard, Lene Christiansen, Anders L. Nielsen, Anders Børglum, Ole Mors

**Affiliations:** 1 Department of Biomedicine, University of Aarhus, Aarhus C, 8000 Denmark; 2 Translational Neuropsychiatric Unit, Aarhus University Hospital, Risskov, 8240 Denmark; 3 Research Department P, Aarhus University Hospital, Risskov, 8240 Denmark; 4 The Lundbeck Foundation Initiative for Integrative Psychiatric Research, iPSYCH, Aarhus C, Denmark; 5 Center for Integrative Sequencing, iSEQ, AU, Aarhus C, Denmark; 6 Department of Public Health, University of Southern Denmark, Odense C, 5000 Denmark

**Keywords:** DNA methylation, Archival dried blood spots, MeDIP-seq, Low input, Genome-wide

## Abstract

**Background:**

In utero and early-life experienced environmental exposures are suggested to play an important role in many multifactorial diseases potentially mediated through lasting effects on the epigenome. As the epigenome in addition remains modifiable throughout life, identifying specific disease-relevant biomarkers may prove challenging. This has led to an increased interest in epigenome-wide association studies using dried blood spots (DBS) routinely collected in perinatal screening programs. Such programs are in place in numerous countries around the world producing large and unique biobanks. However, availability of this biological material is highly limited as each DBS is made only from a few droplets of blood and storage conditions may be suboptimal for epigenetic studies. Furthermore, as relevant markers may reside outside gene bodies, epigenome-wide interrogation is needed.

**Results:**

Here we demonstrate, as a proof of principle, that genome-wide interrogation of the methylome based on methylated DNA immunoprecipitation coupled with next-generation sequencing (MeDIP-seq) is feasible using a single 3.2 mm DBS punch (60 ng DNA) from filter cards archived for up to 16 years. The enrichment profile, sequence quality and distribution of reads across genetic regions were comparable between samples archived 16 years, 4 years and a freshly prepared control sample.

**Conclusions:**

In summary, we show that high-quality MeDIP-seq data is achievable from neonatal screening filter cards stored at room temperature, thereby providing information on annotated as well as on non-RefSeq genes and repetitive elements. Moreover, the quantity of DNA from one DBS punch proved sufficient allowing for multiple epigenome studies using one single DBS.

**Electronic supplementary material:**

The online version of this article (doi:10.1186/s13148-016-0242-1) contains supplementary material, which is available to authorized users.

## Background

Epigenetic regulation has proven important in numerous cellular mechanisms including cell differentiation, gene expression and genome stability [1, 2]. With mounting evidence of epigenetic variations playing an important role in the etiology of common complex diseases such as cancer, diabetes and psychiatric disorders, the interest in epigenome-wide association studies (EWAS), specifically with regard to DNA methylation, has increased extensively in recent years [3–6]. However, identification of epigenetic risk variants with small effect sizes in a heterogenic population requires sample sets of considerable sizes. Additionally, given the modifiable nature of the epigenome distinguishing causality from consequence can prove challenging in adult samples, as marks dynamically change according to cellular environment and differentiation but also as a consequence of adverse exposures and disease. In fact, exposures experienced prenatally have shown to be able to inflict, even long-lasting, epigenetic changes. For example, maternal smoking or stress during pregnancy has shown to result in aberrant epigenetic changes in the offspring [7–9]. In addition, the inherent plasticity of the epigenome leads to changes not only over time per se but also in response to the use of certain pharmaceuticals and other potentially “epitoxic” substances. For instance, DNA methylation changes have been associated with the use of anti-psychotic drugs such as clozapine and haloperidol [10, 11]. A variety of common diseases including type II diabetes and multiple sclerosis have been suggested to include a component of in utero origin [12, 13]. The epigenome is especially vulnerable at this stage, laying the foundation for the “developmental origins of health and disease hypothesis”, suggesting that in utero experienced exposures affect the epigenome [14]. Interrogation of the epigenetic profile is, therefore, preferably done before disease onset and ideally perinatally.

Screening for metabolic disorders in newborns is routinely undertaken in many countries, where blood from heel-pricks is collected on filter paper cards, also known as Guthrie cards. Subsequent storage of the cards essentially produces a population-wide biobank. Notable, since prenatal exposures are believed to have impact on multiple cell lineages, mirror effects may arise. Hence, methylome profiling in DNA extracted from dried blood spot (DBS) samples can potentially be informative both in the search for biomarkers and to delineate the etiology of diseases manifested in other tissues [15, 16]. Also, whereas the intra-individual variation in the epigenetic landscape is considerable, the inter-individual variation is relatively small, making not only longitudinal but also cross-individual studies feasible [17].

Nested case-control approaches taking advantage of large biobanks containing DBS filter cards can potentially satisfy both abovementioned needs of large and early-life sample sets provided that the epigenome remains stable under the storage conditions of the particular biobank and that sufficient biological material can be obtained. Previous studies have shown that DNA can be extracted from DBSs stored at −20°C for decades without considerable loss of quality paving the way for genome-wide association studies (GWAS) and whole genome sequencing (WGS) [18, 19]. Along the same line, both the use of methylation arrays and methylome-wide sequencing using DNA extracted from DBSs has previously shown to be feasible, however, requiring whole genome amplification of bisulfite converted DNA or large input amounts [20–24]. However, amplification of the bisulfitome (bisulfite-converted genomic DNA) has proven to introduce biases [25, 26]. Methylated DNA immunoprecipitation coupled with next-generation sequencing (MeDIP-seq) is a cost-effective methylome alternative, where methylated genomic DNA is immunoprecipitated with an antibody followed by sequencing of the enriched fragments. MeDIP-seq displays good positive correlation with array-based methods such as the HumanMethylation450 BeadChip [21, 27] and also provides information on non-RefSeq genes and repetitive elements, both of which may be of importance in disease development and not interrogated with current methylation arrays [28, 29]. The method of choice is purpose dependent. Arrays may be the appropriate choice for disease studies searching for common variants, whereas MeDIP-seq provides agnostic information on the methylation profile and can be used for a broader characterization of cell development and the impact of environmental exposure outside annotated genomic regions. The minimum DNA input requirements by the different methods is another limiting factor in the case of biobanked DBS filter cards (Additional file 1: Figure S1). With 50 ng of DNA sufficient for MeDIP-seq, this method is one of the least consuming whole genome approaches.

Storage conditions vary among the biobanks, thus, where the Danish National Screening Biobank (DNSB), the California Research-Ready Biospecimen Bank store the filter cards at around −20°C, the Swedish PKU-Biobank, the Michigan Neonatal Biobank and the Danish Twin Registry store the majority of their samples at room temperature.

In this study, we profiled the methylome of DNA stored on room temperature archived filter cards by the use MeDIP-seq using a single 3.2 mm punch. A freshly prepared filter card served as benchmark control. Despite prolonged (4–16 years) storage at room temperature the methylome on a global level appeared largely unchanged. These results underline that DNA extracted from only a small punch of an invaluable archival DBS on a filter card, can be used for genome-wide methylome analyses including methylome-wide association studies (MWAS).

## Results

### MeDIP sequencing quality from DBS samples

Genomic DNA was extracted from one 3.2 mm punch from each of the three Whatman 903 filter cards (Table 1), being a freshly prepared “homemade” sample (hDBS), a short-term (4 years at room temperature) stored sample (rDBS) and a long-term (16 years at room temperature) stored sample (oDBS). All three are independent adult samples from two males and one female. Employing a purpose-specialized protocol, DNA was extracted in triplicates at three separate occasions, routinely yielding on average ~13 ng/mm^2^ (average yield in ng/mm^2^; hDBS 12.4 (±2.4 SD), rDBS 13.4 (±2.6 SD), oDBS 15.1 (±5.3 SD)) (Additional file 2: Figure S2). The DNA was sonicated to a mean fragment length of 180 bp (Additional file 3: Figure S3). Sequencing of MeDIP enriched libraries yielded on average ~68 million clean reads (Additional file 4: Table S1). Sequencing statistics revealed that all three samples performed well, with mean Phred scores above 30 along the entire read, and the anticipated GC content skewedness (Additional file 5: Figure S4).

**Table 1:** Sample overview

Samples	ID	Gender	Storage	Disk size	DNA extracted	MeDIP
1	hDBS (“homemade dried blood spot”)	Male	1 month −20 °C	2.3 mm	111 ng	70 ng
2	rDBS (“recent dried blood spot”)	Female	4 years RT	2.3 mm	94 ng	60 ng
3	oDBS (“old dried blood spot”)	Male	16 years RT	2.3 mm	93 ng	60 ng

Sample description and the amount of genomic DNA extracted and used for MeDIP

*RT* room temperature

**Fig. 1:**
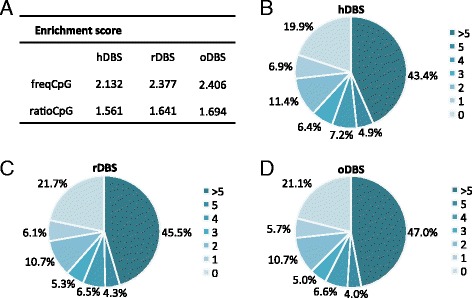
Sequencing quality. **a** Enrichment of CpG sites shown as the calculated frequency of CpGs (fregCpG) and ratio of CpGs (ratioCpG) in the three samples (hDBS, rDBS, oDBS) compared to the reference genome. **b**-**d** Methylome-wide coverage depicted as the percentage of the methylome (28 million CpGs) covered by the sequenced reads in the three samples (hDBS, rDBS, oDBS)

Clean reads were aligned to the human genome (hg38) using Burrows-Wheeler aligner (BWA) algorithm after which duplicated and unmapped reads were removed resulting in a final mean library of ~57 (±2) million mapped reads. Quality and validity check of the mapped MeDIP-seq data was performed using MEDIPS excluding sex chromosomes. Saturation plots showed that all three sets of reads had sufficient complexity and depth to saturate the coverage profile of the reference genome and that this was reproducible (Additional file 6: Figure S5A–C). Assessing the enrichment of methylated regions showed that the relative frequency of CpGs (freqCpG) and the observed/expected ratio of CpGs (ratioCpG) in the genomic regions sequenced compared to the reference genome were above 1 in all cases, indicating a successful enrichment of methylated fragments in the data sets (Fig. 1a). Along this line, the normalization of read counts to CpG density followed the expected linear trajectory, i.e. increased read counts as a function of increased CpG density at CpG poor regions and a deflating relationship at CpG rich regions, which is likely explained by the tendency of CpG islands to be unmethylated (Additional file 6: Figure S5D–E). In the end, 94 ± 2 % of the mapped reads overlapped with a least one CpG, whereas 20.8 ±0.8 % of the in total ~28 million CpGs interrogated were not covered. Conversely, 49.6 ±1.3 % of all CpGs were covered at least five times (Fig. 1b–d). As an additional sequencing quality control for poor alignment and incorrectly mapped reads, visual inspection of the repeat-rich gene *SFI1* was performed (Additional file 7: Figure S6).

### Correlation between the three DBS samples revealed no global changes in methylation patterns

**Fig. 2:**
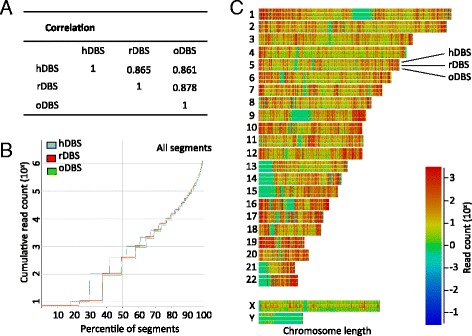
Global methylome comparison. **a** Three-way Pearson’s correlation between hDBS, rDBS and oDBS. **b** Segment trend plot depicting the accumulated number of reads (log transformed) as a function of all segments ranging from the lowest to the highest covered percentile for each of the three samples (hDBS, rDBS and oDBS). **c** Genome-wide domainogram showing the absolute number of all segments in the hDBS, rDBS and oDBS samples along the chromosomes using a color scheme from no segments (*green*) to multiple segments (*dark red*)

With the intend to assess the large scale global differences in DNA methylation patterns in DBS samples as a consequence of prolonged storage at ambient temperatures, a pair-wise Pearson’s correlation was performed between the three DBS samples using MEDIPS excluding sex chromosomes. Since all DBS samples had good saturation profiles it was expected that the read coverage profiles were rather similar although cell composition and genetic variation was unaccounted for. The correlation coefficient ranged from 0.86 to 0.88, indicating that the samples overall had, even without correction for sex specific autosomal DNA methylation differences and age [17, 30], a very similar distribution of reads along the genome and thus an overall similar methylation profile (Fig. 2a, Additional file 8: Figure S7). The degree of correlation falls within a range observed in previous inter-individual methylome profiling studies, especially considering the whole genome MeDIP-seq approach [31–35]. Whereas this suggests that there are no gross changes in the methylation profile, it does not imply that there are no loci with genetic or environmental driven methylation differences.

We looked in greater detail at the distribution of reads along genomic segments defined as 500 bp sliding windows with an overlap of 250 bp. The correlation was normalized only to the total number of reads and log transformed. Cumulating the number of reads from the lowest covered percentile of the genome to the highest should be largely identical between sets of the same type despite defined regional differences such as differently methylated regions (DMRs). The cumulative paths for all three sets appeared much alike, suggesting that no major technically or biologically introduced differences were present, such as large uncovered regions (Fig. 2b). The inconsistency at the lower end of the plot can likely be attributed to the low number of absolute counts of reads. Depicting the read density along the chromosomes in a domainogram again indicated that no systematic bias was evident comparing the three DBS samples. That is, the regional density of reads per segment indicated by a continuum from green (read deserts) to red is overall identical (Fig. 2c). As a check for biological validity, the X chromosome in rDBS (female) had a higher read density as expected, due to X chromosome inactivation, compared to hDBS and oDBS (both males). In addition, no coverage on the Y chromosome in the rDBS sample was observed, as expected.

### Canonical DNA methylation patterns at defined genomic regions

**Fig. 3:**
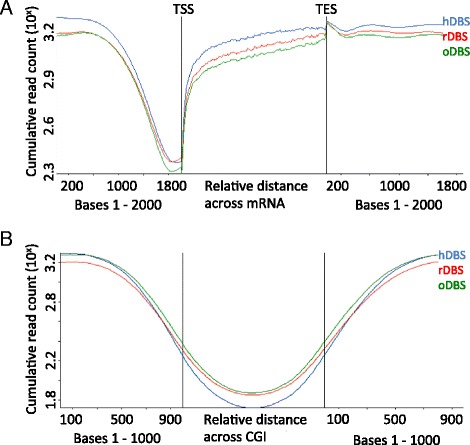
Comparison of the methylation pattern at transcription start site and CpG islands. Read count of 500 bp sliding segments (250 bp overlap) across genetic regions corresponding to **a** mRNA ±2 kb on either side and **b** CGI ±1 kb on either side. *TSS* transcription start site, *TES* transcription end site

An additional quality control step included a more detailed investigation of the specific methylation patterns around known genomic features. This was achieved by calculating the average read count over regions of interest using the 500 bp sliding windows with a 250 bp overlap. Profiling over mRNA transcribed genomic regions ±2 kb showed that the pattern was largely indistinguishable between the three sets. Moreover, a sharp and canonical drop in methylation level around transcription start sites (TSSs) was evident as well as a gradual increase in the methylation throughout the gene body (Fig. 3a). Likewise, displaying mean read count over CpG islands (CGIs) ±1 kb illustrated an overall hypomethylation at CGIs (Fig. 3b).

About one-third of the genomic DNA methylation occurs in repetitive elements such as retrotransposons [36]. Thus, analysis of the methylation level in such elements can be viewed as a proxy for the global genomic methylation status. Bisulfite pyrosequencing of three promoter CpG sites within the highly repetitive long interspersed element 1 (LINE-1) was performed on all three samples (Additional file 9: Figure S8). The LINE-1 CpG sites are normally heavily methylated to prevent retrotransposition. In all our DSB samples, all three sites were highly methylated and within the range of previously reported LINE-1 methylation levels in blood, approximately 80 % [32, 37].

### No apparent systematic change in methylation observed between the DBS samples when clustering based on significant different segments

**Fig. 4:**
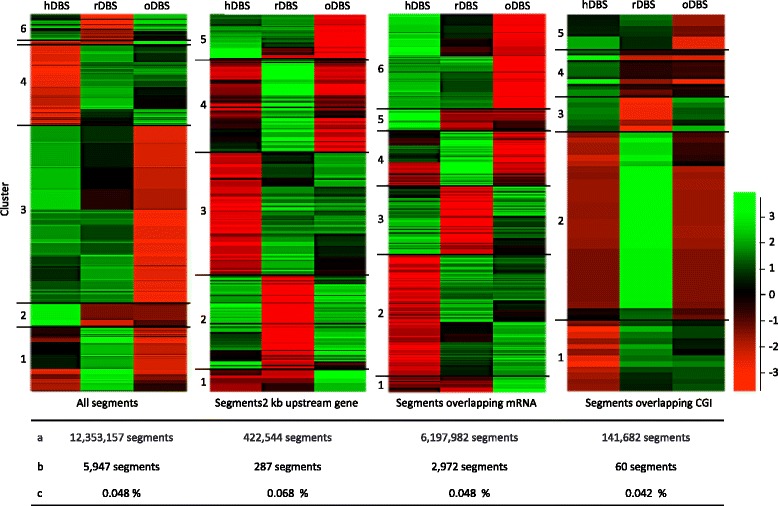
Clustering of segments with significantly different coverage. Three-way unsupervised correlation based clustering of 500 bp segments with a significantly different coverage between at least two of the three samples. Hierarchical clusters are shown with *R* > 0.7. The segments are per-segment normalized (actual segment values minus the median value of that segment-set across all data stores). (*A*) Total number of segments in category; (*B*) number of segments in cluster.; (*C*) percentage of cluster containing segments of all segments in category

With the intent to look at regions with significant variation in methylation levels between the three DBS samples, intensity differences with an multiple testing adjusted *p* value below 0.05 was calculated in a three-way comparison for 500 bp segments individually (Fig. 4a). Of the 1.2 × 10^7^ segments, 5947 (0.05 %) were significantly different between at least two of the three DBS samples and did not appear to aggregate at specific genomic locations (Additional file 10: Figure S9). Segments were grouped to cover promoter regions defined as a 2 kb stretch upstream of the TSS, segments overlapping with mRNA transcribed regions and CGIs. A percent-wise equivalent number of significantly different segments was found in each group. Hence, of the 4.2 × 10^5^ segments covering promoter regions, 287 (0.07 %) were significantly different. Of these, 74 were located on the sex chromosomes.

An unassisted hierarchical clustering of the significantly different segments of each genomic region did not show a clear systematic grouping. There was no general tendency of reduced read density in the archived DBSs compared to the control. Subdividing the clusters, based on a threshold for correlation set to 0.7, divided the cluster into five-six groups. In none of the groups did the segments aggregate at specific genomic locations.

### Only few differentially methylated windows are common for the archived DBS samples

**Fig. 5:**
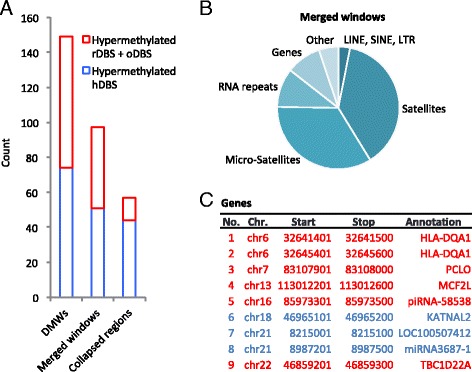
Differentially methylated windows. **a** Bar chart displaying windows of 100 bp with significantly different coverage between hDBS and the merged sample set rDBS + oDBS individually (149), merged if adjacent (97), collapsed if ≤10 kb apart (57). **b** Subdivision of merged windows into genetic categories. **c** Chromosomal position and description of the intragenic merged windows. Marked in *red* indicates hypermethylated in the combined rDBS + oDBS sample set and marked in *blue* hypermethylated in hDBS. HLA-DQA1: Major histocompatibility complex, class II, DQ alpha 1; PCLO: Piccolo presynaptic cytomatrix protein; MCF2L: MCF.2 cell line derived transforming sequence like; KATNAL2: katanin p60 subunit A like 2; TBC1D22A : TBC1 domain family member 22A; LOC100507412: uncharacterized long non-coding RNA LOC100507412; piRNA-58538: piwiRNA 58538; miRNA3687-1: microRNA 3687-1

To further analyze the occurrence of systematic changes to the methylome upon storage, we calculated the differentially methylated windows between hDBS and a merged rDBS/oDBS set using MEDIPS excluding sex chromosomes. Applying an adjusted *p* value ≤0.1 returned 149 significant differently methylated windows, which where reduced to 97 after merging of adjacent windows. A further collapse of the 97 regions spaced less than 10 kb apart produced 57 loci (Fig. 5a). Even at this relaxed threshold with increased risk of false-positives, a modest amount of significant differently windows were detected. For comparison, a more stringent threshold with *p* value ≤0.01 generated 70 significant differently methylated windows. Annotating the 97 merged windows showed that about three-quarters were located in intergenic regions, whereas ten windows were located in rRNA gene clusters and nine intragenic of which three were hypermethylated in hDBS (Fig. 5b, c).

## Discussion

Retrospective methylome studies based on DBSs from biobanked filter cards hold great promise but are potentially hampered by poorer DNA quality and limited availability. In this study, we show that prolonged storage of DBSs, even at room temperature, has little overall influence on the methylome and that methylome-wide coverage can be obtained using DNA extracted from a single 3.2 mm punch, allowing for multiple studies on the same DBS. It has previously been shown that 50 ng of input DNA is sufficient for generating methylation libraries with adequate complexity using MeDIP-seq [38]. A further decrease to 1 ng input is possible, but at the expense of an increased rate of duplicates and immunoprecipitation bias, which leads to loss of data and potentially false-positive findings [22, 39].

From single 3.2 mm DBS punches, we consistently extracted good quality DNA and with no quantitatively loss following prolonged storage, emphasizing the robustness of the extraction methodology applied. In the agnostic search for epigenetic markers or risk variants, a genome-wide approach such as MeDIP may prove valuable. Importantly, DNA extracted in this work proved adequate for MeDIP-seq. The genomic distribution of reads and coverage showed a high positive correlation between the three DBS samples. Moreover, the expected sharp and canonical drop in methylation level around the transcription start sites, as well as the gradual increase in methylation throughout the gene body was evident in all samples [40, 41].

A limitation of the study is that the compared filter cards were not from the same individual. The samples could also not be matched for sex or age [42, 43]. A follow-up study with more and better-matched samples is, therefore, warranted. Despite this, we were able to show that the overall methylation pattern is maintained in DNA isolated from old filter cards stored at room temperature and that this good quality of data can be obtained using MeDIP-seq on low amounts of DNA input. The analysis showed that only very few regions (<0.05 %) differed significantly between at least any two of the three DBS samples. Suggesting that the level of technical or storage-introduced noise is limited. Furthermore, although the three DBS samples originate from different individuals, and thus is not a longitudinal study, the comparison did not reveal any systematic change over the time stored.

Combining the archived DBS samples into one sample set and comparing this to the freshly prepared DBS defined only 149 significant 100 bp windows indicating that even without removal of confounding effects the difference appears limited. The majority of the significant windows were located in repetitive regions. This may be attributed not only to high biological variance at these sites, especially in pericentromeric satellites and subtelomeric elements, but also to alignment issues [44–46]. A previous inter-individual comparison based on MeDIP-seq and peripheral blood monocytes found differences to be highly enriched in repetitive elements, and especially in satellites [33]. Pyrosequencing of three CpGs in the retrotransposable element LINE-1, a surrogate marker for global wide DNA methylation status, showed no apparent difference between the three DBS samples. This may again indicate that an overall loss of methylation has not taken place in the archived DBS samples.

It is important to note that defining true environment-specific or inter-individual DMRs requires that cell type composition and the genetic profile are taken into account. Means of cell composition correction in silico has been devised for array-generated data based on cell type specific methylation patterns [47, 48]. On the other hand, incorporation of genotyping data permits exploration of methylation quantitative trait loci (mQTLs), being a measure of the relationship between genetic polymorphisms and methylation [21]. Notably, a considerable overlap of mQTLs across-tissue has been documented [15, 49]. Also, this allows for examination of gene and environment interactions and the effects on the epigenome [50].

## Conclusions

In conclusion, we demonstrated that the methylome profile is highly comparable between DNA from archived DBS samples and DNA from a freshly prepared DBS sample. Moreover, we show, to our knowledge, for the first time that MeDIP-seq can be performed on neonatal screening cards even with small input amounts. Together, our findings add to the notion that archival DBS samples can be used for sequencing-based epigenome studies, thereby holding great potential in epidemiological research.

## Methods

### Guthrie cards

hDBS: Approximately 70 μL freshly drawn peripheral blood was spotted on Whatman 903 Protein Saver cards (GE Healthcare Life Sciences, Piscataway, NJ, USA). The cards were dried and stored at −20°C.

rDBS/oDBS: Randomly selected from anonymous DBS samples stored at room temperature for 4 years (rDBS) and 16 years (oDBS), respectively. For both, peripheral blood was spotted on the Whatman 903 Protein Saver cards by means of a finger prick.

### DNA extraction

Discs were punched in triplicates from the DBSs by the use of a puncher. Samples stored at RT were incubated in 180 μl PBS at 4 °C overnight in order to increase DNA extraction yield. Following the overnight incubation, PBS was removed and all samples were subjected to the DNA extraction protocol. DNA extraction was performed according to a previously published protocol [51], with introduction of additional centrifugation and ethanol washing steps. Concentration of the extracted DNA was measured using the Qubit instrument and the Qubit dsDNA HS Assay Kit (Thermo Fisher Scientific, Cleveland, OH, USA).

### MeDIP-seq

Extracted genomic DNA (gDNA) was sonicated in a Pico Bioruptor (Diagenode, Seraing, Belgium) at 10 ng/μL for 13 cycles of 30 s on 30 s off to a mean fragment size of 180 bp. Fragment length distribution was assessed by microelectrophoresis using the Qiaxcel instrument and a high-resolution gel cartridge (Qiagen, Hilden, Germany). Sonicated gDNA (in the range of 93–111 ng) was further used for end-repair and adaptor ligation employing the NEBNext Ultra DNA Library Prep Kit for Illumina (New England Biolabs, MA, USA). The reaction mix was purified using Ampure XP beads (Beckman-Coulter, CA, USA) after which methylated DNA immunoprecipitation (MeDIP) was set up on the SX-8G-IP-Star Compact robot (Diagenode) according to manufacturer’s instructions applying the Auto MeDIP kit (Diagenode) and including unmethylated and methylated spike-in controls. Effectively between 60 and 70 ng of adaptor-ligated DNA was used in the MeDIP process. Antibody incubation was performed at 4 °C for 15 h. Immunoprecipitated samples were magnetically purified on the robot using the Auto iPure v2 kit (Diagenode) according to the manufacturer’s instructions. Recovery and enrichment was evaluated by qPCR using primer sets specific for the spike-in controls. Minimum criteria were set to 10 % recovery and 25-fold enrichment. Based on the recovery rate, samples were PCR-amplified at 13 cycles (oDBS) or 14 cycles (hDBS and rDBS) using multiplex oligos (New England Biolabs, MA, USA). Samples were size-selected with Ampure XP beads on the SX-8G-IP-Star Compact robot (Diagenode) using a total of 90 μL beads per sample and eluted in 25 μL DNase-free H_2_O. Purity and fragment length distribution was evaluated on the Qiaxcel instrument. Post-amplification enrichment was verified by qPCR using primer pairs targeting the endogenous hypermethylated promoter region of testis specific histone 2B (*TSH2B*) (Diagenode, cat.nr. C17011041) or the hypomethylated TSS of glyceraldehyde 3-phosphate dehydrogenase (*GAPDH*) (Diagenode, cat.nr. C17011047).

### Sequencing and bioinformatics

Samples were PE50 sequenced on a single lane on a HiSeq2000 instrument (Illumina, CA, USA), generating around 64–71 million clean reads per sample. The reads were cleaned using Soapnuke 1.5.0 tool (BGI, Shenzhen, China) applying the following filters: remove reads with adaptors, remove reads with >10 % unknown reads and remove reads with >50 % low quality bases (*Q* score <5). Clean reads only qualify if Q20 ≥ 85 %.

Using the Galaxy platform, clean reads were groomed and aligned to the human genome (build hg38) using BWA. SAM files were converted to BAM files and filtered to include only de-duplicated uniquely mapped reads resulting in ~54–58 million reads per sample. BAM files were imported to R and SeqMonk v0.32.1 (Barbraham Institute). The R package MEDIPS was used for quality control, genomic coverage estimation and differential coverage analysis [52]. In SeqMonk segments were generated by quantitation of read counts in genetic windows corrected for total counts and with duplicates ignored. Filters include subdivision based on defined features (e.g. genes and CGIs) on any strand and calculating significantly different segments in pair-wise comparisons with a minimum intensity difference *p* value threshold of 0.05 with multiple testing correction (Bonferroni) applied. UCSC Genome Browser was used for visualization and interpretation of genomic regions.

### Pyrosequencing

Genomic DNA from two 3.2 mm DBS punches were pooled and bisulfite-treated using the EpiTect Bisulfite Kit (Qiagen) following the manufacturer’s protocol. A 146 bp fragment of the LINE-1 promoter was amplified by PCR from 100 ng bisulfite-treated DNA and preprocessed for sequencing using the PyroMark Q24 LINE-1 kit (Qiagen) following the manufacturer’s instructions. Prepared PCR products were sequenced on a PyroMark Q24 Advanced and analyzed on the appertaining software (Qiagen) according to the manufacturer’s instructions.

## Abbreviations

BWA, Burrows-Wheeler aligner; CGI, CpG Island; DBS, dried blood spots; DMR, differentially methylated region; DNSB, Danish Neonatal Screening Biobank; EWAS, epigenome-wide association study; GWAS, genome-wide association study; LINE-1, long interspersed nuclear element 1 ; MeDIP, methylated DNA immunoprecipitation; mQTLs, methylation quantitative trait loci; MWAS, methylome-wide association study; TSS, transcription start site; WGS, whole genome sequencing

## Additional files

Additional file 1: Figure S1. Number of experiments achievable per DBS by different methods. Part of a DBS necessary to meet the minimum input requirements of commonly used DNA methylation assessing methods based on an average yield of 13 ng DNA/mm^2^ DBS. (PDF 38 kb) Additional file 2: Figure S2. Robust extraction of genomic DNA from hDBS, rDBS and oDBS. Box-whisker plot depicting median and 1.5 interquartile range (IQR) of gDNA extractions in triplicates at three independent runs for all three filter cards hDBS, rDBS and oDBS. (PDF 42 kb) Additional file 3: Figure S3. Capillary gel electrophoresis electropherograms. DBS extracted DNA was sonicated to a mean length of ~180 bp (upper left). Final hDBS, rDBS and oDBS MeDIP libraries assessed for fragment length distribution and purity. Region of interest mark the targeted length interval of 200 to 500 bp. (PDF 239 kb) Additional file 4: Table S1. Filtering applied to raw reads. Raw reads were filtered by removal of reads i) containing adaptor sequence (“filter adaptor”), ii) with ambiguous bases (≥10 % N per read; “filter N”) and iii) with poor quality (≥50 % bases with *Q* score <5; “filter low quality”). For a sample to qualify the *Q* score must be ≥20 for ≥85 % of the reads. (PDF 31 kb) Additional file 5: Figure S4. Quality of sequence data: (**A**–**C**) Data output overview. (**D**–**F**) Sequencing quality (Q) score across the PE50 reads. (**G**–**I**) GC content in the MeDIP enriched samples (*red line*) compared to the theoretical distribution (*blue line*). (**A**, **D**, **G**) oDBS, (**B**, **F**, **H**) rDBS, and (**C**, **F**, **I**) hDBS. (PDF 619 kb) Additional file 6: Figure S5. Sequencing quality. (**A**–**C**) Saturation analysis indicating adequate complexity and reproducibility of the mapped reads in the hDBS, rDBS and oDBS sample sets compared to the reference genome. (**D**–**F**) Calibration plot showing correct normalization of reads per window in the hDBS, rDBS and oDBS sample sets as a function of CpG density (chromosome 1 only). (PDF 380 kb) Additional file 7: Figure S6. Segment distribution along the repeat-rich gene *SFI1.* Visualization of 500 bp segments (250 bp sliding window) at the *SFI1* locus for hDBS, rDBS and oDBS. (PDF 113 kb) Additional file 8: Figure S7. Pearson’s correlation analysis of hDBS, rDBS and oDBS. Scatter plot depicting the pair-wise Pearson’s correlation of the genome-wide coverage (log transformed number of reads) of hDBS, rDBS and oDBS. (PDF 124 kb) Additional file 9: Figure S8. Pyrosequencing of three promoter CpGs of LINE-1. (**A**) Table listing methylation percentage at each CpG. (**B**) Representative diagram of a single hDBS pyrosequencing reaction. All reactions were performed in triplicate and data is shown as mean ± SD. (PDF 89 kb) Additional file 10: Figure S9. Domainogram of all significantly different segments genome. Domainogram showing the genomic distribution of segments constituting the “all segments” hierarchical cluster. (PDF 859 kb)
