# Supplementary material for: Genome-wide DNA methylation profiling with MeDIP-seq using archived dried blood spots
Source: Clin Epigenetics. 2016 Jul 26;8:81. doi: 10.1186/s13148-016-0242-1 (PMC4960904; doi:10.1186/s13148-016-0242-1)
Supplement: Additional file 1: Figure S1. — Number of experiments achievable per DBS by different methods. Part of a DBS necessary to meet the minimum input requirements of commonly used DNA methylation assessing methods based on an average yield of 13 ng DNA/mm2 DBS. (PDF 38 kb) [file 13148_2016_242_MOESM1_ESM.pdf]

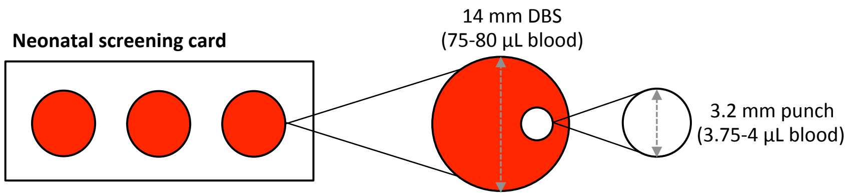

Method	Coverage	Min. DNA requirement	Part of DBS
WGBS-seq	Whole-genome	5 µg	2.5/1
MeDIP-seq	Whole-genome	0.05 µg	1/40
MBD-seq	Whole-genome	1 µg	1/2
RRBS-seq	Genome-wide	0.01 µg	1/200
MRE-seq	Genome-wide	0.5 µg	1/4
450K array	Genome-wide	0.02 µg	1/100
Epityper	Candidate	0.025 µg / amplicon (multiple CpG sites)	1/80
iPlex	Candidate	0.1 µg / plex (~10 CpG sites)	1/20
Pyrosequencing	Candidate	0.01 µg / amplicon (multiple CpG sites)	1/200
