# Supplementary figures and images for: Genome-wide DNA methylation profiling with MeDIP-seq using archived dried blood spots
Source: Clin Epigenetics. 2016 Jul 26;8:81. doi: 10.1186/s13148-016-0242-1 (PMC4960904; doi:10.1186/s13148-016-0242-1)

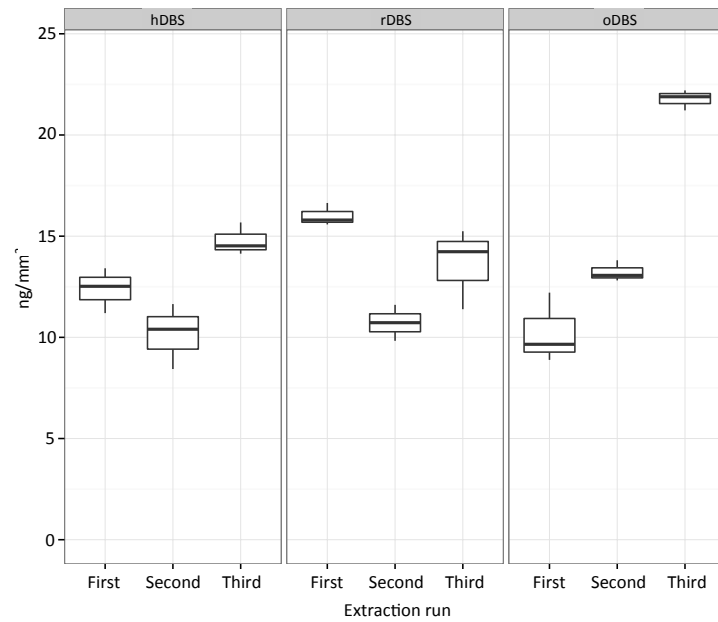

Supplement: Additional file 2: Figure S2. — Robust extraction of genomic DNA from hDBS, rDBS and oDBS. Box-whisker plot depicting median and 1.5 interquartile range (IQR) of gDNA extractions in triplicates at three independent runs for all three filter cards hDBS, rDBS and oDBS. (PDF 42 kb) [file 13148_2016_242_MOESM2_ESM.pdf]

## Supplementary Figure S3

NH Staunstrup et al. 2015

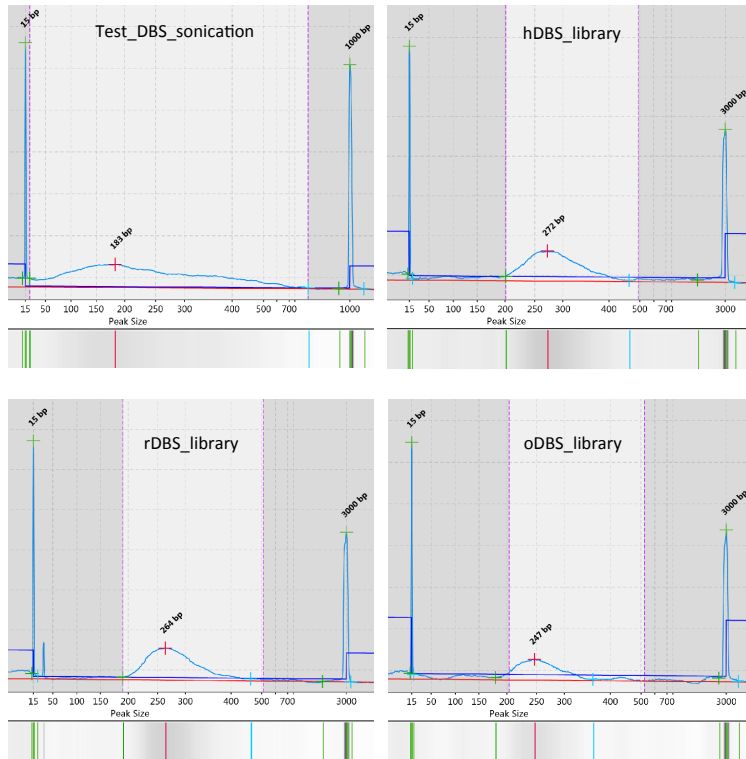

Supplement: Additional file 3: Figure S3. — Capillary gel electrophoresis electropherograms. DBS extracted DNA was sonicated to a mean length of ~180 bp (upper left). Final hDBS, rDBS and oDBS MeDIP libraries assessed for fragment length distribution and purity. Region of interest mark the targeted length interval of 200 to 500 bp. (PDF 239 kb) [file 13148_2016_242_MOESM3_ESM.pdf]

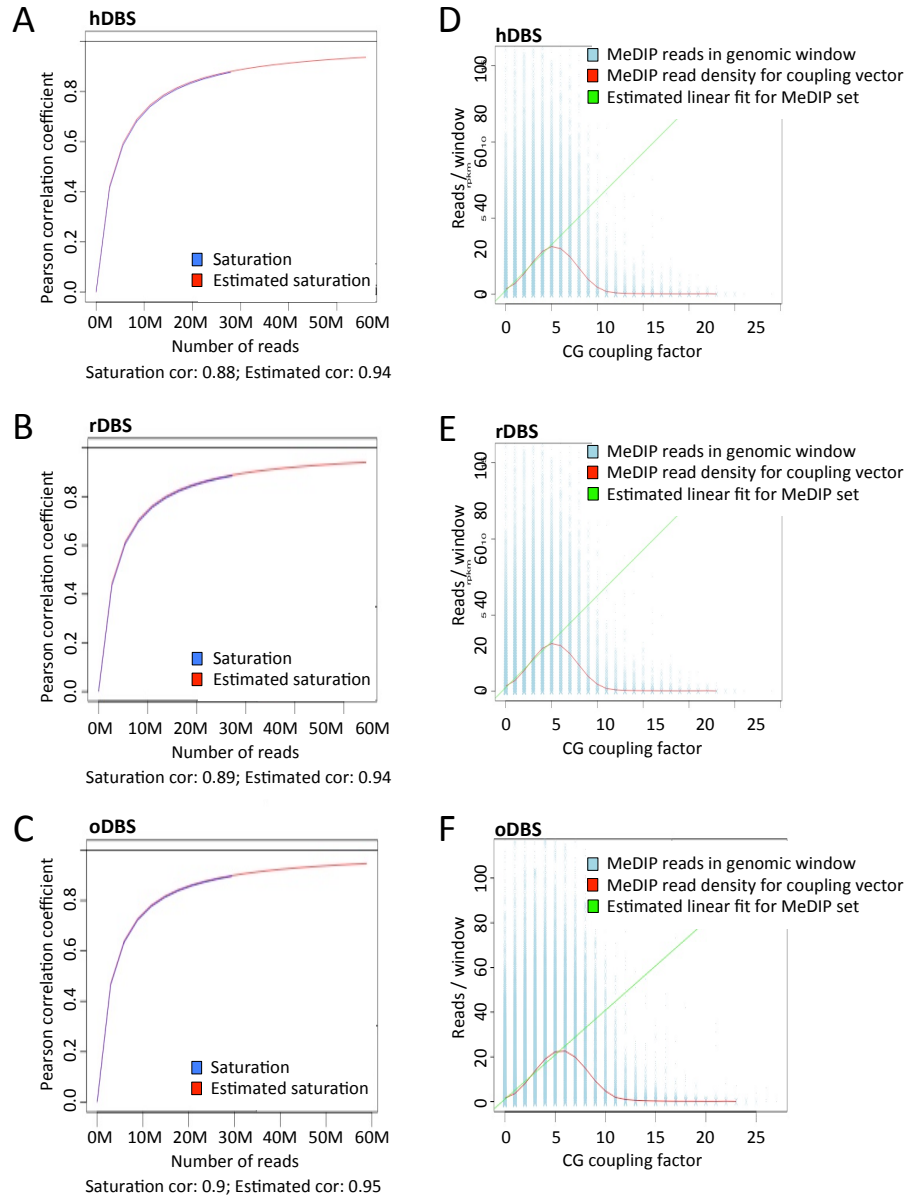

Supplement: Additional file 6: Figure S5. — Sequencing quality. (A–C) Saturation analysis indicating adequate complexity and reproducibility of the mapped reads in the hDBS, rDBS and oDBS sample sets compared to the reference genome. (D–F) Calibration plot showing correct normalization of reads per window in the hDBS, rDBS and oDBS sample sets as a function of CpG density (chromosome 1 only). (PDF 380 kb) [file 13148_2016_242_MOESM6_ESM.pdf]

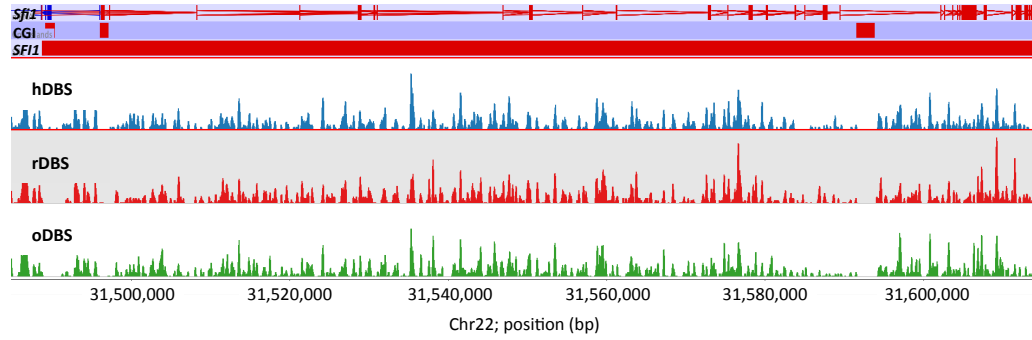

Supplement: Additional file 7: Figure S6. — Segment distribution along the repeat-rich gene SFI1. Visualization of 500 bp segments (250 bp sliding window) at the SFI1 locus for hDBS, rDBS and oDBS. (PDF 113 kb) [file 13148_2016_242_MOESM7_ESM.pdf]

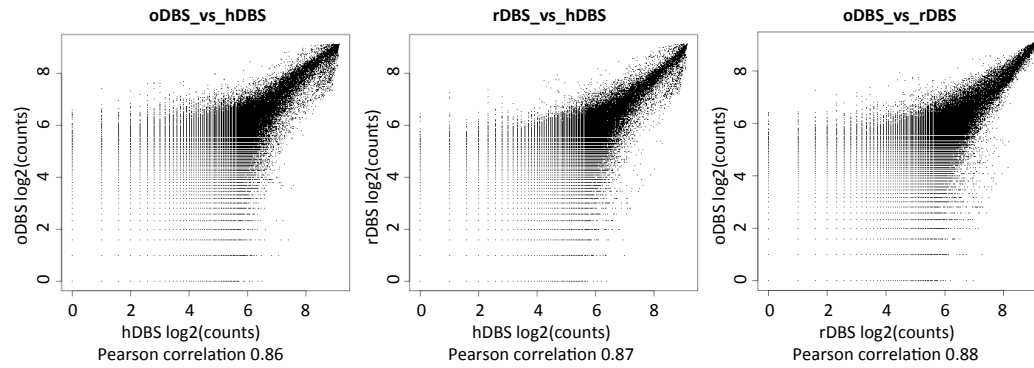

Supplement: Additional file 8: Figure S7. — Pearson’s correlation analysis of hDBS, rDBS and oDBS. Scatter plot depicting the pair-wise Pearson’s correlation of the genome-wide coverage (log transformed number of reads) of hDBS, rDBS and oDBS. (PDF 124 kb) [file 13148_2016_242_MOESM8_ESM.pdf]

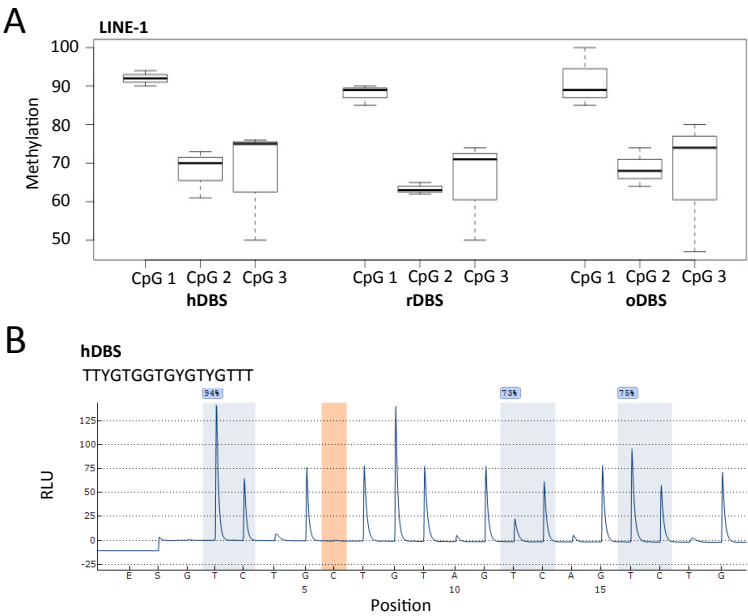

Supplement: Additional file 9: Figure S8. — Pyrosequencing of three promoter CpGs of LINE-1. (A) Table listing methylation percentage at each CpG. (B) Representative diagram of a single hDBS pyrosequencing reaction. All reactions were performed in triplicate and data is shown as mean ± SD. (PDF 89 kb) [file 13148_2016_242_MOESM9_ESM.pdf]

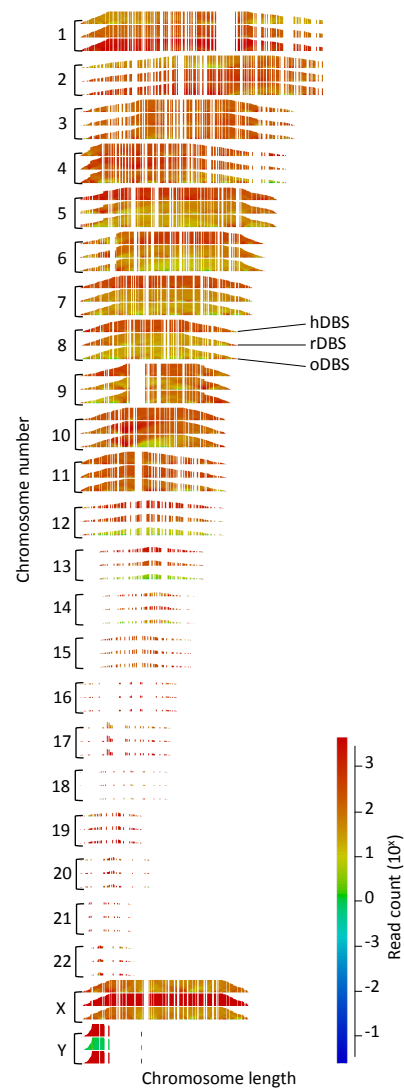

Supplement: Additional file 10: Figure S9. — Domainogram of all significantly different segments genome. Domainogram showing the genomic distribution of segments constituting the “all segments” hierarchical cluster. (PDF 859 kb) [file 13148_2016_242_MOESM10_ESM.pdf]
