# Supplementary material for: Genome-wide DNA methylation profiling with MeDIP-seq using archived dried blood spots
Source: Clin Epigenetics. 2016 Jul 26;8:81. doi: 10.1186/s13148-016-0242-1 (PMC4960904; doi:10.1186/s13148-016-0242-1)
Supplement: Additional file 4: Table S1. — Filtering applied to raw reads. Raw reads were filtered by removal of reads i) containing adaptor sequence (“filter adaptor”), ii) with ambiguous bases (≥10 % N per read; “filter N”) and iii) with poor quality (≥50 % bases with Q score <5; “filter low quality”). For a sample to qualify the Q score must be ≥20 for ≥85 % of the reads. (PDF 31 kb) [file 13148_2016_242_MOESM4_ESM.pdf]

Supplementary Table S1

NH Staunstrup et al. 2015

Sample ID	Total Reads nt	%Q20 before Filter	%Q20 after Filter	%GC before Filter	%GC after Filter	Filter Adapter	Filter N	Filter Low Quality	Clean Reads nt
hDBS	7.01G	96.85%	97.78%	44.70%	44.74%	0.14%	0.32%	1.87%	6.85G
rDBS	6.45G	96.94%	97.82%	46.51%	46.54%	0.13%	0.32%	1.76%	6.31G
oDBS	7.16G	97.12%	97.87%	45.92%	45.93%	0.21%	0.32%	1.37%	7.03G
