# Supplementary material for: Genome-wide DNA methylation profiling with MeDIP-seq using archived dried blood spots
Source: Clin Epigenetics. 2016 Jul 26;8:81. doi: 10.1186/s13148-016-0242-1 (PMC4960904; doi:10.1186/s13148-016-0242-1)

Supplementary Figure S4

NH Staunstrup et al. 2015

A

Basic sequencing statistics

Measure	Value
Filename	*oDBS_2.fq.gz*
File type	Conventional base calls
Encoding	Illumina 1.5
Total sequences	71707275
Seq. flagged as poor quality	0
Sequence length	49
%GC	45

B

Basic sequencing statistics

Measure	Value
Filename	*rDBS_2.fq.gz*
File type	Conventional base calls
Encoding	Illumina 1.5
Total sequences	64374403
Seq. flagged as poor quality	0
Sequence length	49
%GC	46

C

Basic sequencing statistics

Measure	Value
Filename	*hDBS_2.fq.gz*
File type	Conventional base calls
Encoding	Illumina 1.5
Total sequences	69864069
Seq. flagged as poor quality	0
Sequence length	49
%GC	44

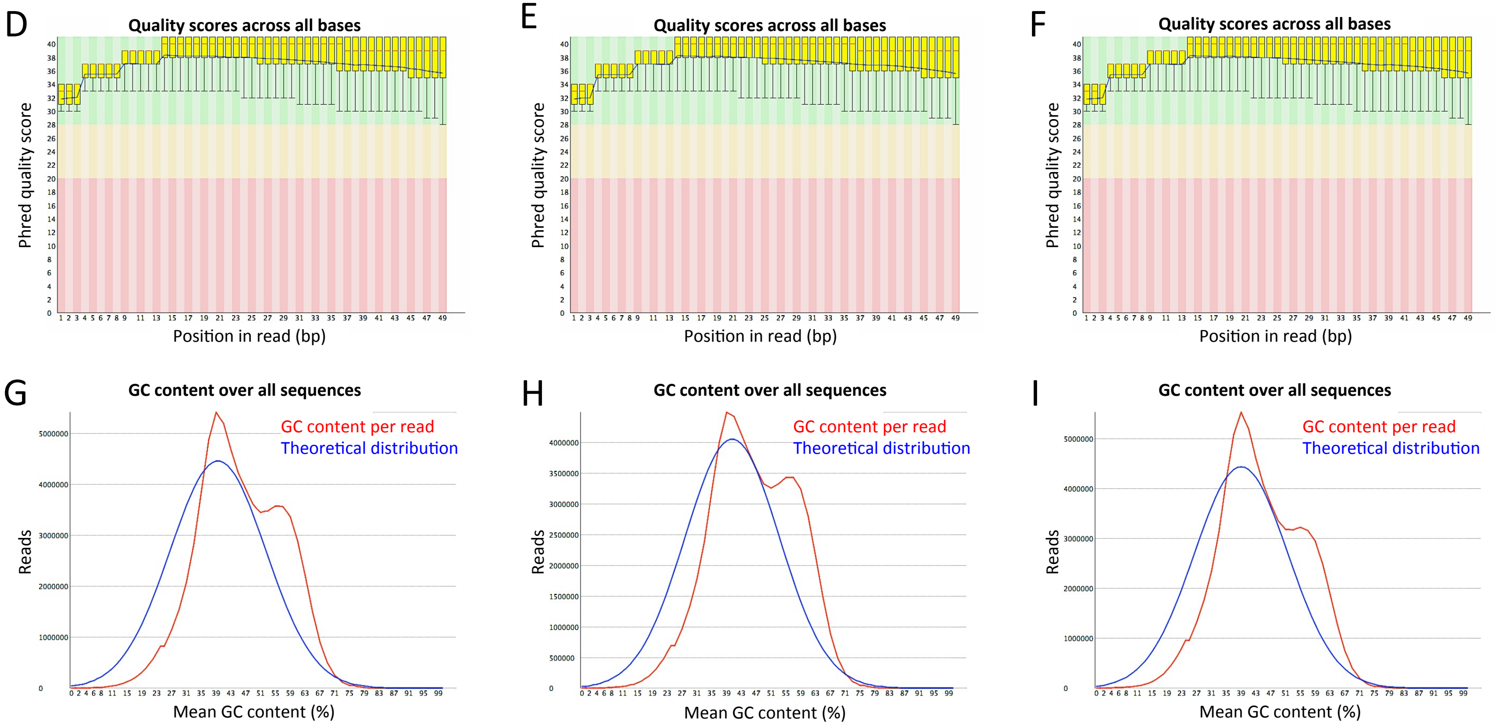

Supplement: Additional file 5: Figure S4. — Quality of sequence data: (A–C) Data output overview. (D–F) Sequencing quality (Q) score across the PE50 reads. (G–I) GC content in the MeDIP enriched samples (red line) compared to the theoretical distribution (blue line). (A, D, G) oDBS, (B, F, H) rDBS, and (C, F, I) hDBS. (PDF 619 kb) [file 13148_2016_242_MOESM5_ESM.pdf]
